# Phase I study of docetaxel in combination with cyclophosphamide as first-line chemotherapy for metastatic breast cancer

**DOI:** 10.1038/sj.bjc.6600626

**Published:** 2002-11-04

**Authors:** P A Vasey, H Roché, D Bisset, C Terret, L Vernillet, A Riva, C Ramazeilles, N Azli, S B Kaye, C J Twelves

**Affiliations:** Cancer Research UK Department of Medical Oncology, Glasgow, UK; Institut Claudius Regaud, Toulouse, France; Department of Oncology, Aberdeen Royal Infirmary, Aberdeen, UK; Aventis, Antony, France; Royal Marsden Hospital, Fulton Road, London, UK

**Keywords:** docetaxel, cyclophosphamide, combination chemotherapy, metastatic breast cancer, dose-finding, pharmacokinetics

## Abstract

This phase I was study conducted to establish the maximum tolerated dose, dose-limiting toxicity, and recommended dose of docetaxel in combination with cyclophosphamide as first-line chemotherapy for metastatic breast cancer. Twenty-six patients were treated with cyclophosphamide (600 mg m^−2^, intravenous bolus) followed by docetaxel (60, 75 or 85 mg m^−2^, 1-h intravenous infusion) every 3 weeks. The maximum tolerated dose was docetaxel 85 mg m^−2^ with cyclophosphamide 600 mg m^−2^, the dose-limiting toxicity being febrile neutropenia. Grade 4 neutropenia was experienced by all patients, but was generally brief. Otherwise, the combination was well tolerated with few acute and no chronic non-haematological toxicities of grade 3/4. Activity was observed at all dose levels and disease sites, and the overall response rate was 42% (95% confidence interval 22–61%). The pharmacokinetics of docetaxel were not modified by cyclophosphamide coadministration. These findings establish a recommended dose of docetaxel 75 mg m^−2^ in combination with cyclophosphamide 600 mg m^−2^ every three weeks for phase II evaluation.

*British Journal of Cancer* (2002) **87**, 1072–1078. doi:10.1038/sj.bjc.6600626 www.bjcancer.com

© 2002 Cancer Research UK

## 

Docetaxel (Taxotere™), a semi-synthetic taxoid, is a relatively new chemotherapeutic agent with high activity against metastatic breast cancer. Following first-line treatment with docetaxel, objective response rates of up to 68% have been reported (Chevallier *et al*, 1995; Dieras *et al*, 1996; Fumoleau *et al*, 1996a; Hudis *et al*, 1996). As second-line treatment for patients with disease resistant to anthracyclines, currently the most widely administered chemotherapeutic agents, docetaxel has achieved the highest activity to date (Ravdin *et al*, 1995; Valero *et al*, 1995; Alexopoulos *et al*, 1999; Nabholtz *et al*, 1999; Sjostrom *et al*, 1999).

Since the use of combination chemotherapy has been associated with increased response rates compared to single agents (Fossati *et al*, 1998), a large programme exploring the feasibility and activity of docetaxel, in combination with the most active agents in metastatic breast cancer, was initiated. Several phase I studies were performed, with combinations including docetaxel and doxorubicin (Misset *et al*, 1999), docetaxel+doxorubicin+cyclophosphamide (Nabholtz *et al*, 1997), docetaxel and epidoxorubicin (Pagani *et al*, 1999), docetaxel and cisplatin (Crown *et al*, 1997), docetaxel and vinorelbine (Fumoleau *et al*, 1996b), and docetaxel and 5-fluorouracil (Lortholary *et al*, 1997).

The programme is also exploring the combination of docetaxel and cyclophosphamide. The rationale for this combination is based on the known clinical activity of both docetaxel and cyclophosphamide (Rubens *et al*, 1975) in advanced breast cancer, and results of preclinical studies in a tumour-bearing mouse model showing synergy in the therapeutic response to the two drugs (Bissery *et al*, 1995). In the mouse model, 60% of the highest nontoxic dose of each agent was administered in combination without additional toxicity.

Considering these clinical and preclinical data, we conducted this phase I study to explore the feasibility of docetaxel in combination with cyclophosphamide. The aims of the study were to determine the maximum tolerated dose, the dose-limiting toxicity, and the recommended dose for phase II studies of the combination as first-line chemotherapy for metastatic breast cancer. A secondary objective was to determine the pharmacokinetic profile of docetaxel in the combination. The starting docetaxel dose of 60 mg m^−2^ was selected on the basis of its known antitumour activity and good safety profile when administered as a single agent (Adachi *et al*, 1996). The dose of cyclophosphamide was fixed at 600 mg m^−2^ since this is the effective dose considered active while producing little toxicity in current protocols.

## PATIENTS AND METHODS

### Study design and patients

This was a phase I, open-label, non-randomised, dose-finding study conducted at two centres (Glasgow, UK; Toulouse, France) between May 1994 and August 1996.

The study population consisted of women aged between 18 and 75 years with locally advanced or metastatic breast cancer. Histological or cytological proof of metastasis was required for patients with a single metastatic lesion. Further inclusion criteria were measurable and/or evaluable disease, a WHO performance status⩽2, normal haematological values (neutrophils⩾2×10^9^ l^−1^, platelets⩾100×10^9^ l^−1^, haemoglobin⩾10 g dl^−1^), normal renal function (creatinine⩽140 μmol l^−1^, creatinine clearance 60 ml min^−1^), and normal liver function test (bilirubin⩽1.25 times the upper limit of the institutional normal value [N]; for the highest dose level only [docetaxel 100 mg m^−2^ and cyclophosphamide 600 mg m^−2^], alanine and aspartate aminotransferases⩽3N, alkaline phosphatase⩽5N).

Patients were excluded if they had received previous chemotherapy for metastatic disease. However, prior neoadjuvant and adjuvant treatments were permitted provided there had been a chemotherapy-free interval of at least 6 months before study entry. Hormonal therapies as adjuvant treatment and/or for metastatic disease (⩽2) were also allowed provided there was a 4-week interval between the last hormonal treatment and study entry in patients who achieved a response (no time interval was required for patients with no response). Radiotherapy given at least 4 weeks previously was permitted except at sites used to assess response in this study. For all previous antitumour therapies, patients must have fully recovered from any toxic effects. Patients were ineligible if they had received previous treatment with docetaxel, paclitaxel, or colony-stimulating factors.

Further exclusion criteria were pregnancy, lactation, or child-bearing potential (e.g. not using adequate contraception); a history of prior malignancies, with the exceptions of non-melanoma skin cancer and well excised cervical carcinoma *in situ*; known clinical brain or leptomeningeal involvement; symptomatic peripheral neuropathy of grade ⩾2; other serious illnesses and medical conditions; and participation in another clinical study either during the study or within 30 days of study entry. For the highest dose level (docetaxel 100 mg m^−2^ and cyclophosphamide 600 mg m^−2^), impaired liver function with alanine and aspartate aminotransferases >1.5 N associated with alkaline phosphatase >2.5 N was also an exclusion criterion.

The study was conducted in accordance with the Declaration of Helsinki, in compliance with local regulations, and with the approval of an independent Ethics Committee at each centre. All patients gave written, informed consent to participate in the study.

### Treatment plan

Four escalating dose levels of docetaxel and cyclophosphamide were planned (Table 1

**Table 1:**
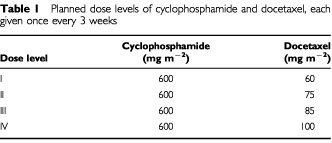
Planned dose levels of cyclophosphamide and docetaxel, each given once every 3 weeks

). Cyclophosphamide was to be administered at a fixed dose (600 mg m^−2^, intravenous bolus) followed by docetaxel, without recombinant human granulocyte colony-stimulating factor (rHuG-CSF) prophylaxis, at increasing doses (60, 75, 85 and 100 mg m^−2^; 1-h intravenous infusion) every 3 weeks in successive groups of patients. The order of drug administration was chosen based on preliminary data from a phase I study examining combination therapy with paclitaxel (135 mg m^−2^ starting dose) and cyclophosphamide (750 mg m^−2^ fixed dose) (Kennedy *et al*, 1993). The study explored both cyclophosphamide followed by paclitaxel and the reverse sequence. The results indicated that haematological toxicity was sequence-dependent: the median neutrophil nadir was lower with paclitaxel given first (964× 10^6^ l^−1^) than with cyclophosphamide given first (1864×10^6^ l^−1^).

To avoid hypersensitivity reactions and docetaxel-related skin toxicity and fluid retention, all patients were given a 3-day corticosteroid prophylactic premedication, starting the day before chemotherapy: oral dexamethasone (20 mg) and ranitidine (150 mg) were administered 3 and 12 h before docetaxel infusion and at 0, 12, 24 and 36 h post-infusion, and oral chlorpheniramine (8 mg) was given at 3 and 12 h before docetaxel infusion.

Treatment was planned for six cycles unless there was evidence of disease progression, unacceptable toxicity, patient refusal, or no symptomatic improvement after three cycles. Treatment thereafter depended on the tumour response during the first six cycles. Patients with an objective response could continue treatment until disease progression, serious toxicity, or patient refusal occurred. Patients with stable disease after six cycles or progression at any time were withdrawn and received salvage treatment at the discretion of the investigator.

Dose levels were assigned at recruitment and no dose escalation was allowed within the same patient. The protocol stipulated that at least three patients should be treated at each dose level, with a 1-week interval between entry of the first patient and the next two patients. Before escalating to the next dose level, at least two patients should have received at least two cycles and have been observed for acute toxicity for a minimum of 2 weeks. If one out of the three patients at the same dose level developed dose-limiting toxicity, three more patients were to be entered at the same dose level. The maximum tolerated dose was defined as the dose level at which at least two out of the six patients developed a dose-limiting toxicity. The recommended dose was defined as the dose level previous to the maximum tolerated dose. For all dose levels tested, more than three patients were recruited, in order to ascertain the safety profile in a larger sample size.

Toxicity was graded according to National Cancer Institute Common Toxicity Criteria (NCI-CTC) current at the start of the study. Dose-limiting toxicities were defined as: grade 4 neutropenia (neutrophil count <0.5×10^9^ l^−1^) for >7 days; febrile neutropenia (grade 4 neutropenia concomitant with grade ⩾2 fever); grade 4 thrombocytopenia (platelet count <25×10^9^ l^−1^); grade 3/4 bleeding or infection; grade 4 nausea/vomiting; grade 2 neurological toxicity; and any other grade 3/4 toxicities, except alopecia and anaemia. Non-recovery of the neutrophil count (to ⩾1.5×10^9^ l^−1^) or platelet count (to ⩾100×10^9^ l^−1^) on day 21 of each cycle were further dose-limiting toxicities.

Dose modifications were planned for haematological and non-haematological toxicity. Except for grade 3 neuropathy, for which patients were withdrawn, in the event of dose-limiting toxicity, treatment was delayed until recovery to grade 1 then restarted usually at the dose level below or modified as appropriate to the toxicity. If, despite the corticosteroid prophylactic premedication, a hypersensitivity reaction developed, the symptoms were treated appropriately, for example with epinephrine for anaphylactic shock and aminophylline for bronchospasm. No dose modification was planned for localised or peripheral oedema, but the patient could be withdrawn at the discretion of the investigator. Antiemetic prophylaxis (metoclopramide or domperidone) could be given from the first cycle onwards, and loperamide to prevent diarrhoea in subsequent cycles following grade 2/3 diarrhoea; patients with nausea or diarrhoea despite these measures could be treated with granisetron or odansetron, but additional treatment with corticosteroids was not allowed.

### Patient and treatment evaluation

Prestudy evaluations included a medical history and physical examination, an electrocardiogram, radiology examinations (bilateral mammography, chest X-ray or CT scan, abdominal ultrasound or CT scan, bone scan, and instrumental examinations as appropriate for any other measurable and/or evaluable disease), a complete blood count (white blood cells with differential, platelets, haemoglobin), a biochemistry profile, a baseline toxicity evaluation, and other investigations as clinically indicated.

Patients were monitored regularly for toxicity, and were asked to report any clinical adverse events to the investigator. Haematological assessments were made twice weekly, or every 2 days in cases of febrile neutropenia until recovery to grade⩽1 neutropenia and resolution of all infectious symptoms. Irrespective of the reason for treatment discontinuation, patients were observed during the first month after the last cycle of treatment in order to monitor any late adverse events.

Tumour response was assessed according to WHO criteria (Miller *et al*, 1981). The duration of a partial response dated from the start of treatment until the first documentation of progressive disease, while the duration of a complete response was from the time it was first documented. The time to first response and time to progression were from the start of study treatment to the first occurrence of response and first progression, respectively. Survival dated from the start of treatment.

### Pharmacokinetic analysis

The pharmacokinetics of docetaxel were evaluated during the first cycle only, whereas the pharmacokinetics of cyclophosphamide were not determined. Blood samples were collected before intravenous infusion of docetaxel, at 30 min after the start of infusion, at the end of infusion, and at 5, 10, 20, 30, 60, 90 min and 2, 4, 6, 8, 12, 16, 24, 36, 48 and 72 h post-infusion. Plasma docetaxel concentrations were measured by high-performance liquid chromatography, using a C18 reversed-phase column and ultraviolet detection at 225 nm (Vergniol *et al*, 1992). The lower quantification limit of this assay was 10 ng ml^−1^. Pharmacokinetic parameters were estimated by nonlinear least square regression analysis using WinNonlin software (Scientific Consulting Inc, USA) and a two- or three-compartment open model with first-order elimination.

The following parameters were calculated: the maximum plasma concentration (C_max_), the area under the plasma concentration-time curve (AUC[0-∞]), total body clearance (CL), the volume of distribution at steady state (V_ss_), and the terminal elimination half-life (t_½z_).

## RESULTS

### Patient characteristics

Twenty-six patients with previously untreated metastatic breast cancer were enrolled into the study (Table 2

**Table 2:**
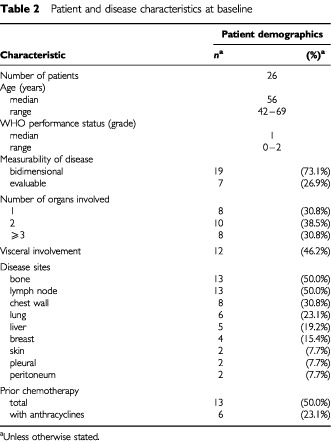
Patient and disease characteristics at baseline

). The median age was 56 years and the median WHO performance status was 1. Eighteen patients (69%) had two or more involved organs; 12 (46%) had visceral involvement, and bone and lymph nodes were the most frequent disease sites (both 13 patients; 50%). Oestrogen receptor status was performed in only nine patients; six were ER negative, three ER positive. Progesterone receptor status was not assessed in any patient. Thirteen patients (50%) had received previous adjuvant and/or neoadjuvant chemotherapy, with an anthracyline-based regimen in six patients (23%). Sixteen patients had received adjuvant hormonal therapy, 16 as treatment for metastatic disease, and nine received hormonal therapy both in the adjuvant and metastatic setting. Nineteen patients (73%) had at least one bidimensionally measurable lesion, while the remaining seven patients (27%) had only evaluable disease.

### Chemotherapy administration

In total, 144 cycles of chemotherapy were administered during the study (Table 3

**Table 3:**
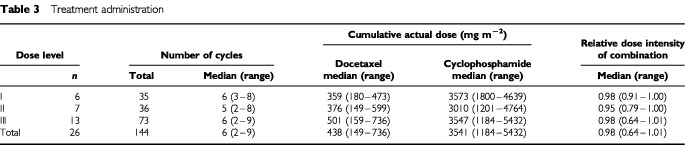
Treatment administration

). The median number of cycles per patient was six (range 2–9). At least four cycles were given to 20 patients (77%), six cycles to 17 patients (65%), and eight cycles to six patients (23%). The median cumulative doses of docetaxel and cyclophosphamide were 438 mg m^−2^ (range 149–736 mg m^−2^) and 3541 mg m^−2^ (range 1184–5432 mg m^−2^), respectively. The median relative dose intensity was 0.98 for each of docetaxel, cyclophosphamide, and the combination.

Most treatment cycles (135; 94%) were administered every 3 weeks as planned. Only nine cycles (6%) were delayed and seven delays lasted for no longer than 1 week. Dose modification was required for six cycles (4%). Five delays and five dose modifications were due to toxicity; the remainder were because of non-medical reasons.

### Safety

All 26 patients were evaluable for safety. Overall, the toxicities seen at all dose levels were usually haematological and of short duration, and were rarely severe or non-haematological. Fourteen patients (54%) discontinued the study after they had completed the planned six cycles of treatment. Of the 12 patients (46%) who discontinued treatment earlier, 10 withdrew due to disease progression; one patient (at the docetaxel 75 mg m^−2^ dose level) withdrew due to moderate fluid etention that was observed at cycle 5 and after a cumulative docetaxel dose of 288 mg m^−2^. One patient at the docetaxel 60 mg m^−2^ dose level died secondary to perforation of the ascending colon complicated by peritonitis. The patient had been taking non-steroidal anti-inflammatory drugs (NSAIDs) in addition to her corticosteroids, and presented with haematemesis. She was neutropenic (0.46×10^9^ l^−1^) but typhlitis was not suspected because of the clinical presentation and the absence of prolonged granulocytopenia. However, a post-mortem examination was not carried out.

As expected with these two myelosuppressive drugs, neutropenia and its complications were the most frequent adverse events (Table 4

**Table 4:**
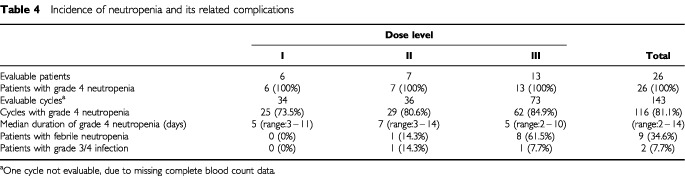
Incidence of neutropenia and its related complications

). Grade 4 neutropenia developed in all patients and its incidence by cycle was highest at the docetaxel 85 mg m^−2^ dose level. The grade 4 neutropenia was, however, generally brief, with a median duration of 5 to 7 days and a median time to nadir of 7 to 8 days across the dose levels. At day 22±3, the median neutrophil count was ⩾4.9×10^9^ l^−1^ at all dose levels. Febrile neutropenia was defined broadly, as grade 4 neutropenia concomitant with grade ⩾2 fever, and occurred in nine patients (35%; 7% of cycles). The highest incidence was observed at the docetaxel 85 mg m^−2^ dose level (eight patients). However, at this dose level, only one patient developed a documented grade 3/4 infection (grade 3 urinary infection at cycle 2).

Although anaemia was noted in all patients (eight grade 1, 14 grade 2, four grade 3), no grade 4 episode occurred. Eight patients (31%) required a blood transfusion during the study. Thrombocytopaenia was uncommon, occurring in four patients (15%), and no patient experienced a grade 3/4 episode or required a platelet transfusion.

The most common acute non-haematological toxicities considered to be possibly or probably related to study treatment were nausea, stomatitis, diarrhoea, and vomiting (Table 5

**Table 5:**
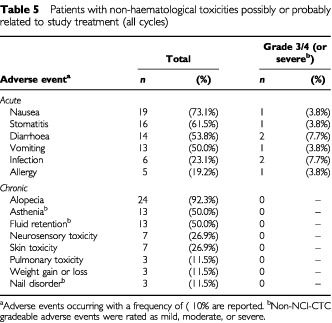
Patients with non-haematological toxicities possibly or probably related to study treatment (all cycles)

). Episodes were usually grade 1/2; only a few grade 3/4 episodes were observed during the study. Alopecia was universal. Asthenia and fluid retention were frequent (each 50% of patients), but were never severe. One patient discontinued the study due to moderate fluid retention. The other docetaxel-related chronic toxicities (i.e. skin toxicity and nail disorders) were infrequent and never severe.

### Maximum tolerated and recommended doses

The maximum tolerated dose of the combination was reached at the docetaxel 85 mg m^−2^ dose level. Of the 13 patients treated at this dose level, six developed dose-limiting toxicity, as a result of febrile neutropenia (five patients) or grade three infection (one patient). The dose-limiting toxicity of the combination was therefore considered to be febrile neutropenia. The recommended dose of the combination for phase II evaluation is cyclophosphamide 600 mg m^−2^ administered as an intravenous bolus followed by docetaxel 75 mg m^−2^ given as a 1 h intravenous infusion.

### Efficacy

Of the 26 patients, only one achieved a complete response and 10 a partial response, giving an overall response rate of 42% (95% confidence interval [CI] 22–61%) (Table 6

**Table 6:**
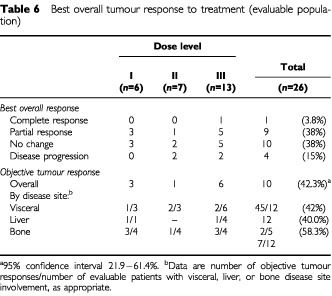
Best overall tumour response to treatment (evaluable population)

). Objective responses occurred at all dose levels and disease sites, but were most common at the docetaxel 85 mg m^−2^ dose level, with five partial responses and one complete response among the 12 evaluable patients. The complete response was achieved at the docetaxel 85 mg m^−2^ dose level by a patient with axillary lymph node involvement, but with no visceral or bone involvement. The time to first response ranged from 2.9 to 19.9+ weeks and the duration of response from 15.0 to 52.3+ weeks. It should be noted that the response lasted for longer than 25 weeks in eight out of the 10 responding patients. The duration of response was censored in one-half of responders, due to further radiotherapy (one patient) and no documentation of progressive disease at the study cut-off date (four patients).

The median time to disease progression was 25.9 weeks (range 4.1 to 55.1 weeks), and with a median follow-up of 7.9 months, median survival was 10.8 months (range: 1.6 to 18.4 months).

### Pharmacokinetics of docetaxel

The pharmacokinetic profile of docetaxel was evaluated in 20 patients. Marked inter-patient variability was observed especially for V_ss_ and t_½z_. However, the C_max_ and AUC[0-∞] of docetaxel increased as expected with dose. The CL, V_ss_, and t_½z_ of docetaxel were relatively stable over the dose range investigated, with mean overall values of 22.1 l h m^−2^, 93 l m^−2^, and 11.8 h, respectively (Table 7

**Table 7:**
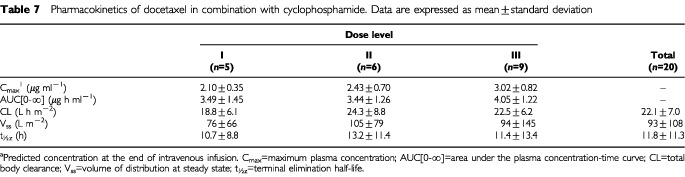
Pharmacokinetics of docetaxel in combination with cyclophosphamide. Data are expressed as mean±standard deviation

).

## DISCUSSION

A wide range of drugs have been tested in breast cancer, and historically the agents with the greatest activity are anthracyclines, alkylating agents, 5-fluorouracil, methotrexate, cyclophosphamide, and vinca alkaloids (Henderson, 1991; Marty *et al*, 1992). The anthracyclines have long since been considered as the most active agents in the treatment of breast cancer, with reported response rates ranging from 29% (second-line) to 53% (first-line) (Frederiksen *et al*, 1978; Steiner *et al*, 1983; Jain *et al*, 1985; Brambilla *et al*, 1986; Carmo-Pereira *et al*, 1987; Richards *et al*, 1992). Combination regimens are more effective than single-agent chemotherapy, and currently the highest overall response rate in breast cancer (up to 60%) is achieved with anthracycline-containing regimens such as cyclophosphamide, doxorubicin or epirubicin, and 5-fluorouracil (CAF or FEC) (Mouridsen, 1992; Harris *et al*, 1993). Doxorubicin is the most widely administered anthracycline. However, continued administration of this agent is limited by an increased risk of cardiotoxicity, and a cumulative dose not exceeding 450–550 mg m^−2^ is recommended (von Hoff *et al*, 1979). Furthermore, anthracycline-based regimens are increasingly being used as adjuvant and/or neoadjuvant chemotherapy for early breast cancer, and anthracylines therefore have a limited role as salvage treatment in patients previously exposed in these settings. There is therefore a clear need to develop new, non-anthracycline-based regimens that are active in patients failing to respond to anthracyclines or who can no longer receive them.

With these considerations, we initiated this phase I study, aiming to define the safety profile and to determine the maximum tolerated dose, the dose-limiting toxicity, and the recommended dose of docetaxel in combination with cyclophosphamide, without colony-stimulating factors, as first-line chemotherapy in patients with metastatic breast cancer.

The study establishes a recommended dose of docetaxel 75 mg m^−2^ in combination with cyclophosphamide 600 mg m^−2^ given every 3 weeks, without the support of prophylactic rHuG-CSF, for phase II evaluation in patients with previously untreated metastatic breast cancer. The maximum tolerated dose was reached at the dose level combining docetaxel 85 mg m^−2^ and cyclophosphamide 600 mg m^−2^, the dose-limiting toxicity being febrile neutropenia.

The finding that febrile neutropenia was the dose-limiting toxicity of docetaxel with cyclophosphamide is in agreement with an earlier dose-finding study evaluating this combination in patients with various advanced solid tumour types, including patients with metastatic breast cancer (Valero, 1997). There were, however, significant differences in study design between this and the earlier study. In contrast with our study, both previously treated and untreated patients were eligible for the earlier study and dose escalation of both agents was planned.

The starting dose was identical in this and the earlier advanced solid tumour study (Valero, 1997) and in both the combination of docetaxel 75 mg m^−2^ and cyclophosphamide 600 mg m^−2^ was well tolerated. Interestingly, further dose escalation was feasible in the earlier study, to docetaxel 75 mg m^−2^ and cyclophosphamide 700 mg m^−2^ for previously treated patients and to docetaxel 75 mg m^−2^ and 800 mg m^−2^ for previously untreated patients. The protocol for our study scheduled further dose escalation, of both docetaxel (85–100 mg m^−2^) and cyclophosphamide (600–1800 mg m^−2^), supported by rHuG-CSF (lenograstim 150 μg m^−2^ day^−1^ from day 2 until neutrophil recovery, defined as neutrophil count ⩾1.0×10^9^ l^−1^), should the dose-limiting toxicity be neutropenia or its complications. Since the dose-limiting toxicity in the initial part of the study was febrile neutropenia, this second-step dose escalation was undertaken (Chollet *et al*, 1998). A preliminary evaluation showed that the use of lenograstim enabled substantial increases in the doses of both agents: the maxi mum tolerated dose was not reached even at the dose level of docetaxel 100 mg m^−2^ in combination with cyclophosphamide 1200 mg m^−2^. Recent studies conducted by the National Surgical Adjuvant Breast and Bowel Project–B-22 (Fisher *et al*, 1997) and B-25 (Fisher *et al*, 1999) – showed no benefit in terms of disease-free or overall survival of increasing the dose intensity and the cumulative dose of cyclophosphamide (with or without rHuG-CSF support) over the standard dose of 600 mg m^−2^ in early breast cancer patients receiving combination doxorubicin and cyclophosphamide chemotherapy. For this reason, we decided to discontinue the study, stopping further escalation to doce- taxel 100 mg m^−2^ in combination with cyclophosphamide 1500 mg m^−2^ or 1800 mg m^−2^.

Febrile neutropenia was the only dose-limiting toxicity in this and the earlier advanced solid tumour study (Valero, 1997). Neutropenia and its complications are also dose-limiting in combination chemotherapy with paclitaxel and cyclophosphamide for advanced or metastatic breast cancer (Kennedy *et al*, 1996; Pagani *et al*, 1997). However, with the paclitaxel and cyclophosphamide combination, severe thrombocytopenia and non-haematological toxicity are also reported as dose limiting toxicities. In patients with advanced breast cancer previously treated with no more than one chemotherapy regimen, dose-limiting toxicities of febrile neutropenia and severe thrombocytopenia defined a maximum tolerated dose of paclitaxel 200 mg m^−2^ with cyclophosphamide 2000 mg m^−2^ with or without rHuG-CSF prophylaxis (Schrijvers *et al*, 1993). For anthracycline-resistant metastatic breast cancer, even with rHuG-CSF prophylaxis, dose-limiting toxicities including myelosuppression, neuropathy, myalgia, and typhilitis defined the maximum tolerated dose of paclitaxel 200 mg m^−2^ with cyclophosphamide 1250 mg m^−2^ (Kennedy *et al*, 1996). Interestingly, in the dose escalation with lenograstim conducted following this study, no dose-limiting toxicity was observed even at the highest administered dose level of docetaxel 100 mg m^−2^ in combination with cyclophosphamide 1200 mg m^−2^.

Fluid retention, a frequent adverse event described in early studies of docetaxel (van Oosterom and Schrijvers, 1995; Schrijvers *et al*, 1993) occurred in one-half of all patients in this study, but was never severe. The reduced incidence and severity of docetaxel-related fluid retention in this and other recent studies is probably due to the routine administration of a 3-day corticosteroid premedication regimen (Riva *et al*, 1997).

Docetaxel 60 mg m^−2^ was chosen as the starting dose in this study on the basis of its antitumour activity and tolerability as a single agent (Adachi *et al*, 1996). It was therefore not surprising that antitumour activity was observed at each docetaxel and cyclophosphamide dose level, and at all disease sites. The overall tumour response rate was rather lower than that achieved in the earlier advanced solid tumour study (42 versus 69%). However, it should be considered that since this was a phase I study, efficacy assessment was not a primary end-point. Only one-quarter of patients had evaluable disease, and one-half had bone metastases, which are difficult to assess. Moreover, due to the small study size, the CI around the response rate of 42% was wide. Phase II studies are needed and planned to further explore the efficacy of the recommended dose of docetaxel 75 mg m^−2^ and cyclophosphamide 600 mg m^−2^.

Although this study defined the recommended dose of docetaxel in combination with cyclophosphamide for phase II studies as 75 mg m^−2^, it is of note that the docetaxel 85 mg m^−2^ dose level was reasonably well tolerated, despite the incidence of dose-limiting febrile neutropenia. Among 13 patients treated at this dose level, there were no deaths and the median relative dose intensity of the combination approached 1. This dose level was the most active (50% overall tumour response rate), and could be considered for further evaluation.

The pharmacokinetics of docetaxel did not appear to be influenced by cyclophosphamide when the two drugs were given as combination chemotherapy, as compared with historical data from studies of docetaxel monotherapy. All parameters were in agreement with those in two phase I studies in which single-agent docetaxel was administered as a 1- to 2-h intravenous infusion at doses ranging from 20–115 mg m^−2^ (Bruno *et al*, 1996). Total body clearance in our study was relatively stable over the three dose levels tested and almost identical to that for single-agent docetaxel (22.1 versus 21.0 l h m^−2^). Interpatient variability in V_ss_ and t_½z_ values was marked, but overall mean values were similar to those described for single-agent docetaxel (V_ss_=93 versus 67 l m^−2^; t_½z_=12 versus 11 h). Docetaxel and cyclophosphamide can therefore be administered together without any relevant drug interaction according to this administration schedule.

In conclusion, docetaxel in combination with cyclophosphamide is a well tolerated and active combination for first-line chemotherapy of metastatic breast cancer. With the increasing use of anthracycline-based chemotherapy as adjuvant therapy for metastatic breast cancer, patients are frequently exposed to high cumulative doses of anthracyclines and are therefore at risk of resistance and cardiotoxicity. The combination of docetaxel with cyclophosphamide, or other non-anthracycline chemotherapeutic agents, may be particularly useful in patients previously treated with anthracycline-based chemotherapy but naïve to the non-anthracycline agents. However, it must be noted that although combination chemotherapy is generally associated with higher response rates in solid tumour oncology, there is no clear evidence for a survival benefit over the administration of single agents in metastatic breast cancer. Moreover, because combination chemotherapy is invariably associated with more toxicity, consideration must be given to the fact that the primary aim of treatment in this situation is palliation. A phase II study utilising the recommended dose of docetaxel 75 mg m^−2^ and cyclophosphamide 600 mg m^−2^ will further explore the activity and confirm the safety of the combination.
